# Supplementary material for: Dynamic control of enhancer activity drives stage-specific gene expression during flower morphogenesis
Source: Nat Commun. 2019 Apr 12;10:1705. doi: 10.1038/s41467-019-09513-2 (PMC6461659; doi:10.1038/s41467-019-09513-2)
Supplement: Supplementary file 1 — Supplementary Information [file 41467_2019_9513_MOESM1_ESM.pdf]

Supplementary Information

Supplementary Figures

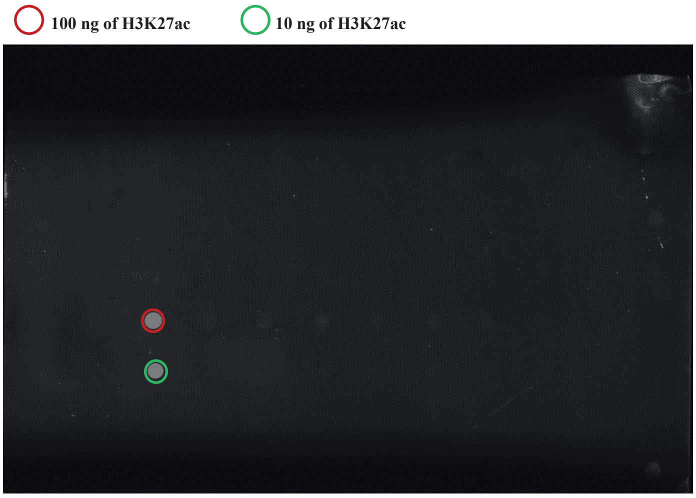

Histone H3 Array

	1	2	3	4	5	6	7	8	9	10	11	12	
A	H3 1-19 unmod	H3 1-19 R2me1	H3 1-19 R2me2a	H3 1-19 R2me2s	H3 1-19 T3P	H3 1-19 K4ac	H3 1-19 K4me1	H3 1-19 K4me2	H3 1-19 K4me3	H3 1-19 R8me1	H3 1-19 R8me2a	H3 1-19 R8me2s	100 ng
B	H3 1-19 unmod	H3 1-19 R2me1	H3 1-19 R2me2a	H3 1-19 R2me2s	H3 1-19 T3P	H3 1-19 K4ac	H3 1-19 K4me1	H3 1-19 K4me2	H3 1-19 K4me3	H3 1-19 R8me1	H3 1-19 R8me2a	H3 1-19 R8me2s	10 ng
C	H3 1-19 K9ac	H3 1-19 K9me1	H3 1-19 K9me2	H3 1-19 K9me3	H3 1-19 S10P	H3 1-19 T11P	H3 7-26 unmod	H3 7-26 K14ac	H3 7-26 R17me1	H3 7-26 R17me2a	H3 7-26 R17me2s	H3 7-26 K18ac	100 ng
D	H3 1-19 K9ac	H3 1-19 K9me1	H3 1-19 K9me2	H3 1-19 K9me3	H3 1-19 S10P	H3 1-19 T11P	H3 7-26 unmod	H3 7-26 K14ac	H3 7-26 R17me1	H3 7-26 R17me2a	H3 7-26 R17me2s	H3 7-26 K18ac	10 ng
E	H3 16-34 unmod	H3 16-34 K23ac	H3 16-34 K27ac	H3 16-34 K27me1	H3 16-34 K27me2	H3 16-34 K27me3	H3 16-34 R26me1	H3 16-34 R26me2a	H3 16-34 R26me2s	H3 16-34 S28P	H3 26-44 unmod	H3 26-44 K36ac	100 ng
F	H3 16-34 unmod	H3 16-34 K23ac	H3 16-34 K27ac	H3 16-34 K27me1	H3 16-34 K27me2	H3 16-34 K27me3	H3 16-34 R26me1	H3 16-34 R26me2a	H3 16-34 R26me2s	H3 16-34 S28P	H3 26-44 unmod	H3 26-44 K36ac	10 ng
G	H3 26-44 K36me1	H3 26-44 K36me2	H3 26-44 K36me3	H3 26-44 Y41P	H3 47-65 unmod	H3 47-65 K56ac	H3 71-89 unmod	H3 71-89 K79me1	H3 71-89 K79me2	H3 71-89 K79me3	Rat IgG 10 ng	Sheep IgG 10 ng	100 ng
H	H3 26-44 K36me1	H3 26-44 K36me2	H3 26-44 K36me3	H3 26-44 Y41P	H3 47-65 unmod	H3 47-65 K56ac	H3 71-89 unmod	H3 71-89 K79me1	H3 71-89 K79me2	H3 71-89 K79me3	Mouse IgG 10 ng	Rabbit IgG 10 ng	10 ng

Supplementary Fig. 1: Validation of the specificity of H3K27ac antibody for immunoprecipitation. Western blot against several H3 specific histone modifications (AbSurance Histone H3 array) was performed to check the specificity of H3K27ac antibody. Only H3K27ac modification but not any of other H3 unmodified peptides gave signal.

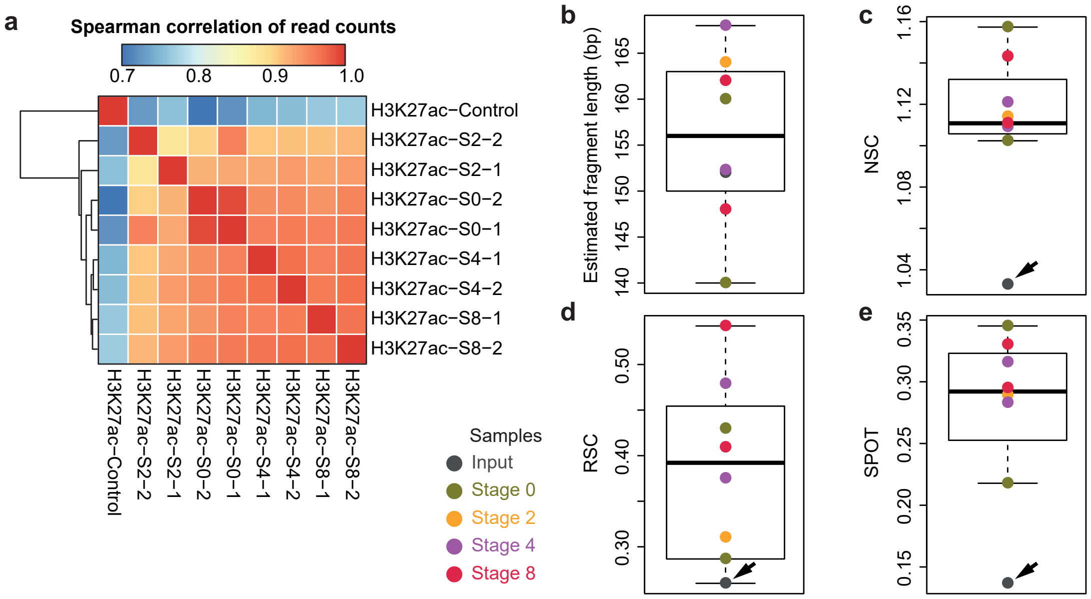

Supplementary Fig. 2: Reproducibility and quality controls of H3K27ac ChIP-seq data. (a) Spearman's correlations of read counts in H3K27ac ChIP-seq replicates. Analysis was performed by deepTools. S0, stage 0. (b-e) Quality controls of the H3K27ac ChIP-seq data using a variety of metrics. Different samples are colored according to sampling stages. Input sample is used for control. (b) Estimated fragment length. Note there is no difference between ChIP samples and the input sample. (c) Normalized strand cross-correlation coefficient (NSC). NSC values range from a minimum of 1 to larger positive numbers. Low value means low signal to noise or few peaks. (d) Relative strand cross-correlation coefficient (RSC). RSC values range from 0 to larger positive values. The low scores can be due to failed and poor quality ChIP, low read sequence quality and hence lots of mismappings, shallow sequencing depth (significantly below saturation) or a combination of these. (e) Signal portion of tags (SPOT). SPOT is a measure of enrichment (analogous to the fraction of reads in peaks) with its value ranging from 0 to 1. SPOT calculates the fraction of reads that fall in tag-enriched regions identified using the Hotspot program from a sample of 5 million reads. Larger SPOT values indicate higher signal to noise.

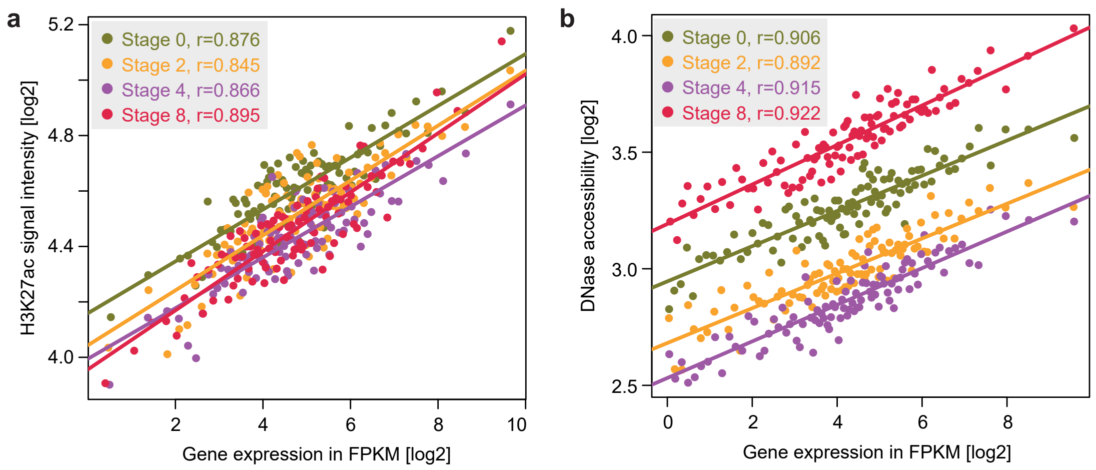

Supplementary Fig. 3: Correlation between gene expression and H3K27ac / DNase-seq intensities (related to Figure 1c). (a) Scatter plot comparing gene expression and H3K27ac binding intensity at promoters. Genes were binned into percentiles (N=100) based on their expression level, and the mean expression and mean binding intensity of each bin were plotted. The x-axis shows gene expression in fragments per kilobase of transcript per million mapped reads (FPKM); the y-axis represents normalized H3K27ac enrichment at promoters. (b) Scatter plot comparing gene expression and promoter accessibility. Representation is similar to (a).

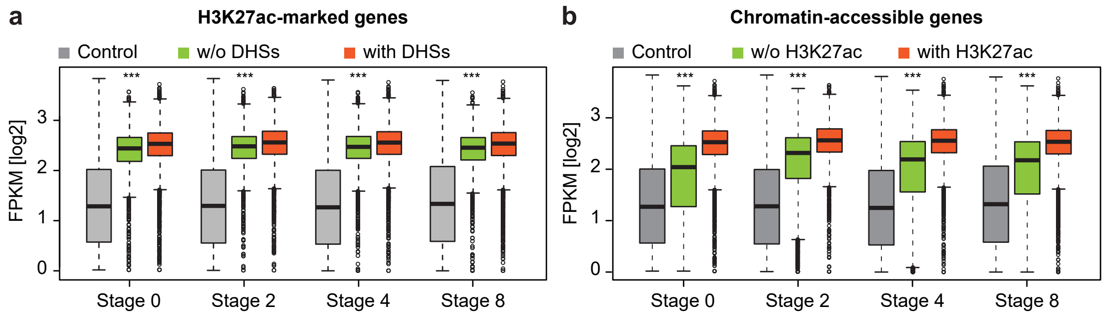

Supplementary Fig. 4: Expression dynamics of H3K27ac-marked or chromatin-accessible genes. (a) Boxplots of gene expression for H3K27ac-marked genes associated with or without (w/o) DNase I hypersensitive sites (DHSs). The control group (colored in gray) indicates the expression pattern of genes without H3K27ac modification and without DHSs. (b) Boxplots of gene expression for chromatin-accessible genes associated with or without (w/o) H3K27ac modification. The control group (colored in gray) indicates the expression pattern of genes without H3K27ac modification and without chromatin accessibility. Boxplots show the median (line), second to third quartiles (box), the interquartile range (whiskers) and outliers (dots). (***), p-value < 0.001, Student's t-test.

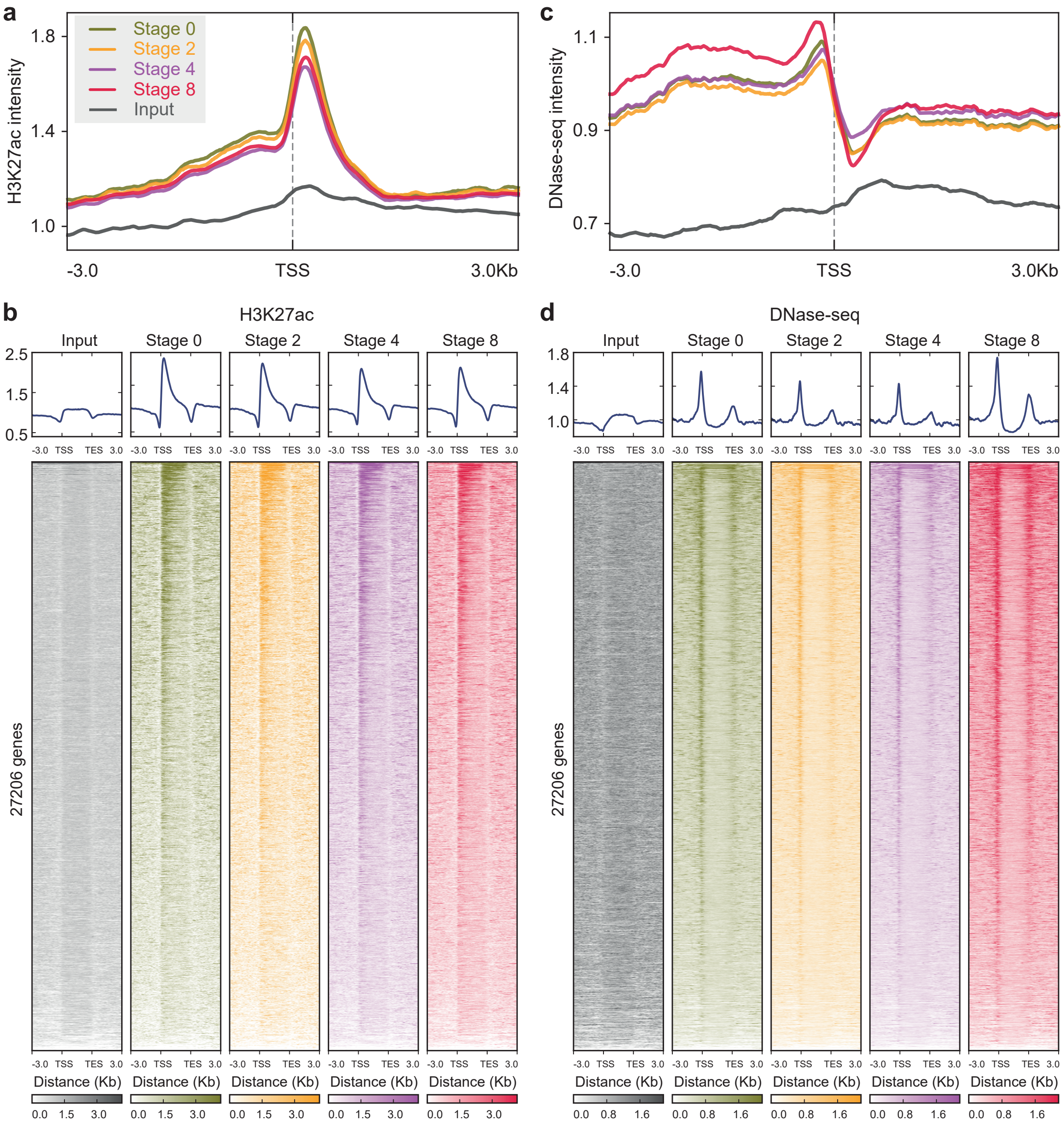

Supplementary Fig. 5: Distribution of H3K27ac ChIP-seq and DNase-seq reads (related to Figure 1c). (a,c) Enrichment of H3K27ac binding signals (a) and chromatin accessibility (c) around the transcription start site (TSS). (b,d) Distribution of H3K27ac ChIP-seq (b) and DNase-seq (d) reads around gene body. TES, transcription termination site.

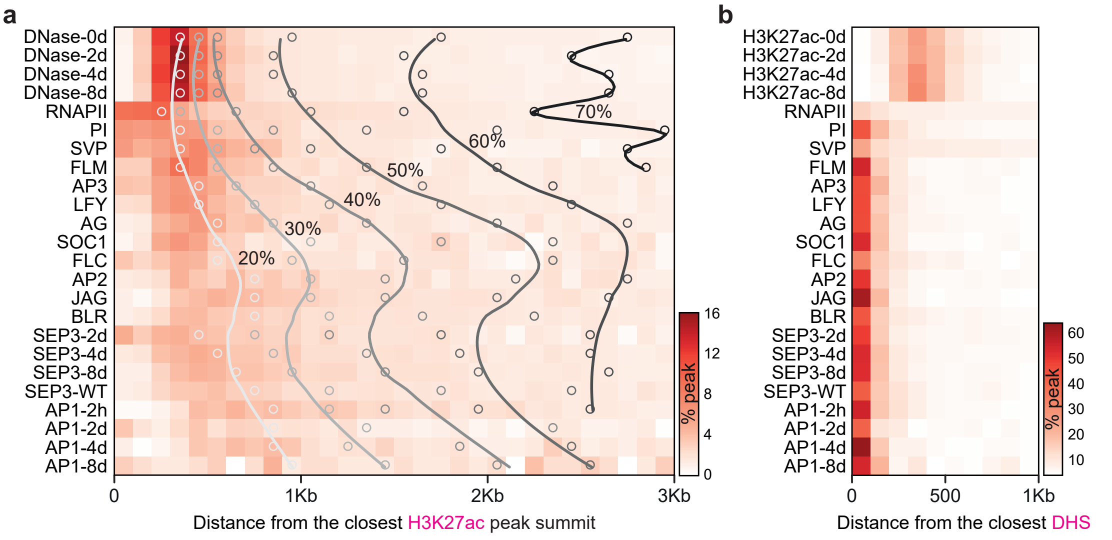

Supplementary Fig. 6: Distribution of transcription factor (TF) binding sites relative to the summit of H3K27ac peaks or DHSs. (a) Calculation of the distance of TF binding sites (TFBSs) relative to their closest H3K27ac sites (summits). Curves denote the accumulative fraction of H3K27ac peaks. (b) Similar analysis based on DHSs. Note that most TFBSs located closely to DHSs but far away from H3K27ac sites.

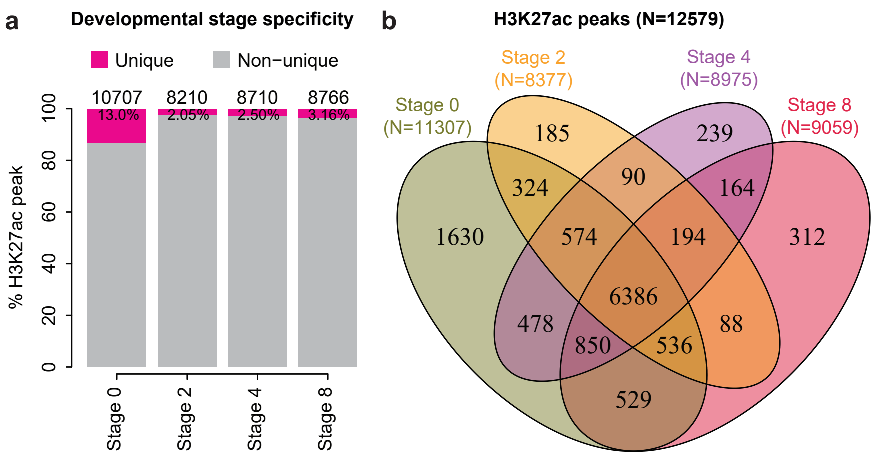

Supplementary Fig. 7: Comparison of H3K27ac peaks between different stages. (a) Stacked bar plot of the unique (stage-specific) and non-unique H3K27ac peaks. The total count of peaks are labeled above the bars. (b) Peaks from all the four stages were firstly merged as a reference set of peaks. The peaks of each stage were overlapped with the reference peaks and were then counted based on the genomic coordinate of reference peaks if any overlapping.

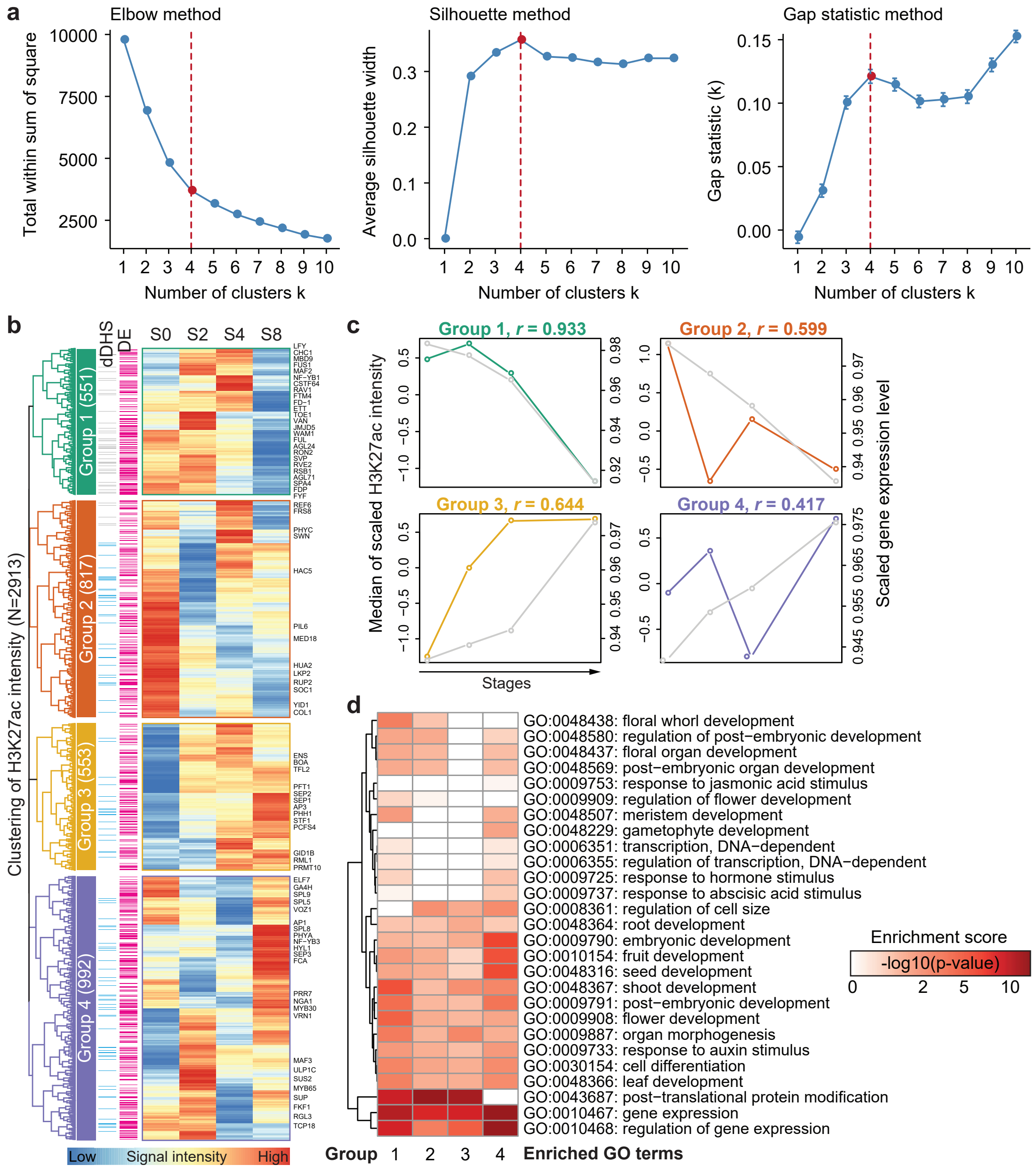

Supplementary Fig. 8: Dynamically changed H3K27ac-associated regions across flower development. (a) Determining the optimal number of clusters. Three different methods (elbow, silhouette and gap statistic) were used to compute the optimal number of clusters (k=4 in all cases as highlighted in red). (b) Heatmap showing the clustering analysis of 2913 H3K27ac peak regions that dynamically change more than 1.5-fold across the four stages (from stages 0 [S0] to 8). These regions are clustered into four groups (in different colors) with distinct patterns of H3K27ac enrichment. The blue bars on the left of heatmap indicate that the nearest DHSs for the corresponding H3K27ac peaks display higher than 1.5-fold change (dDHS). The pink bars indicate the nearest genes are differentially expressed (DE). Flowering genes are labeled on the right of heatmap. (c) Summary (median value) of H3K27ac enrichment in the four groups as colored in (b). Grey curves indicate the median scaled expression level of genes in the corresponding groups. Correlation coefficient values between H3K27ac signal intensity and gene expression are shown. (d) Gene ontology (GO) enrichment analysis for each gene clusters as represented in (b).

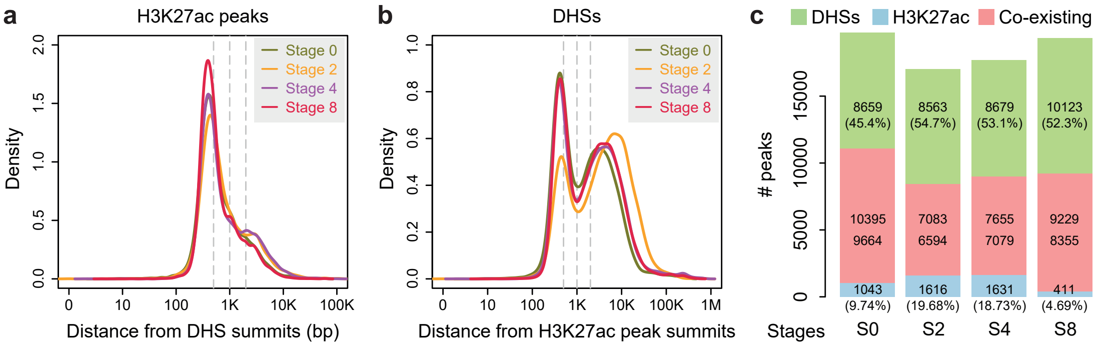

Supplementary Fig. 9: Analysis of H3K27ac sites. (a) Distribution of the distance between H3K27ac sites (midpoints) and their nearest DHS peak summits. (b) Distribution of the distance between DHSs (peak summits) and their nearest H3K27ac midpoints. (c) Bar plot showing the co-existence of H3K27ac peaks and DHSs. An H3K27ac peak and a DHS are considered as coexisting if they resided within 1 kb of each other. Numbers under the co-existence category indicate the number of overlapping regions for DHSs (above) and H3K27ac (below), respectively. Percentage values in parentheses show the percentage of non-coexistence regions.

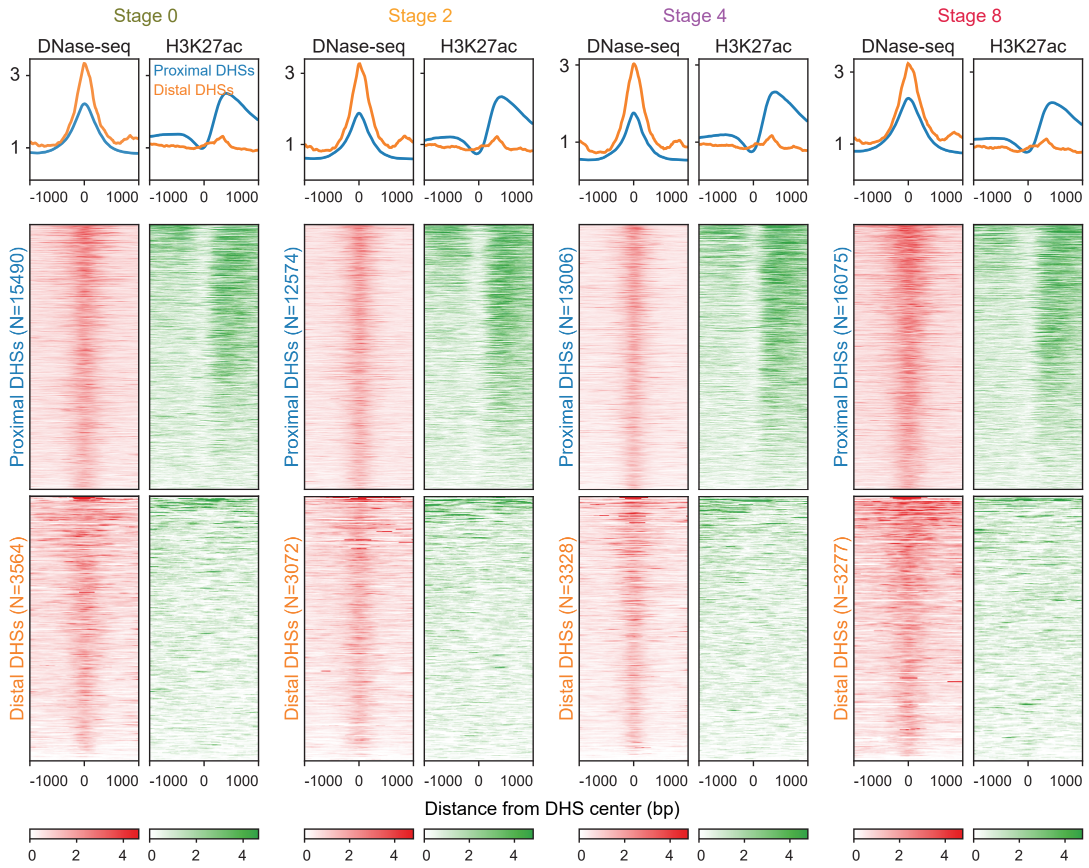

Supplementary Fig. 10: H3K27ac and DNase-seq read intensity relative to proximal and distal DHS midpoints. Above parts, composite (average intensity) plots; below parts, heatmaps of read distribution per DHS.

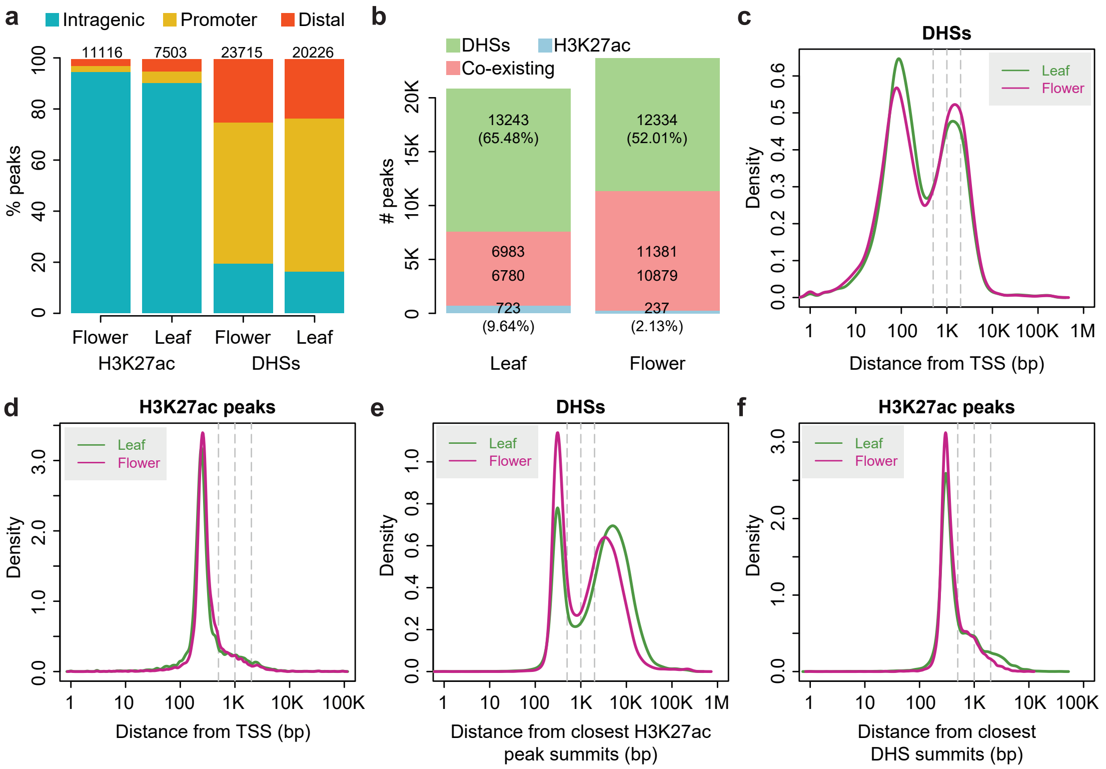

Supplementary Fig. 11: Reanalysis of published H3K27ac ChIP-seq and DNase-seq data from Zhang et al. (a) Stacked bar plot showing the distribution of H3K27ac peaks and DNase I hypersensitive sites (DHSs) in different genomic regions in leaves and flowers. Promoters are defined as genomic regions encompassing 1 kb upstream of the TSS. Distal intergenic regions are genomic regions except intragenic and promoter regions. Peaks were assigned to each category based on their summits. (b) Bar plot showing the co-existence of H3K27ac peaks and DHSs. An H3K27ac peak and a DHS are considered as coexisting if they resided within 1 kb of each other. Numbers under the co-existence category indicate the number of overlapping regions for DHSs (above) and H3K27ac (below), respectively. Percentage values in parentheses show the percentage of non-coexistence regions. (c) Distribution of the distance between DHSs (peak summits) and their closest TSSs. (d) Distribution of the distance between H3K27ac peak summits and their closest TSSs. (e) Distribution of the distance between H3K27ac peak summits and their nearest H3K27ac midpoints. (f) Distribution of the distance between H3K27ac sites (midpoints) and their nearest DHS peak summits.

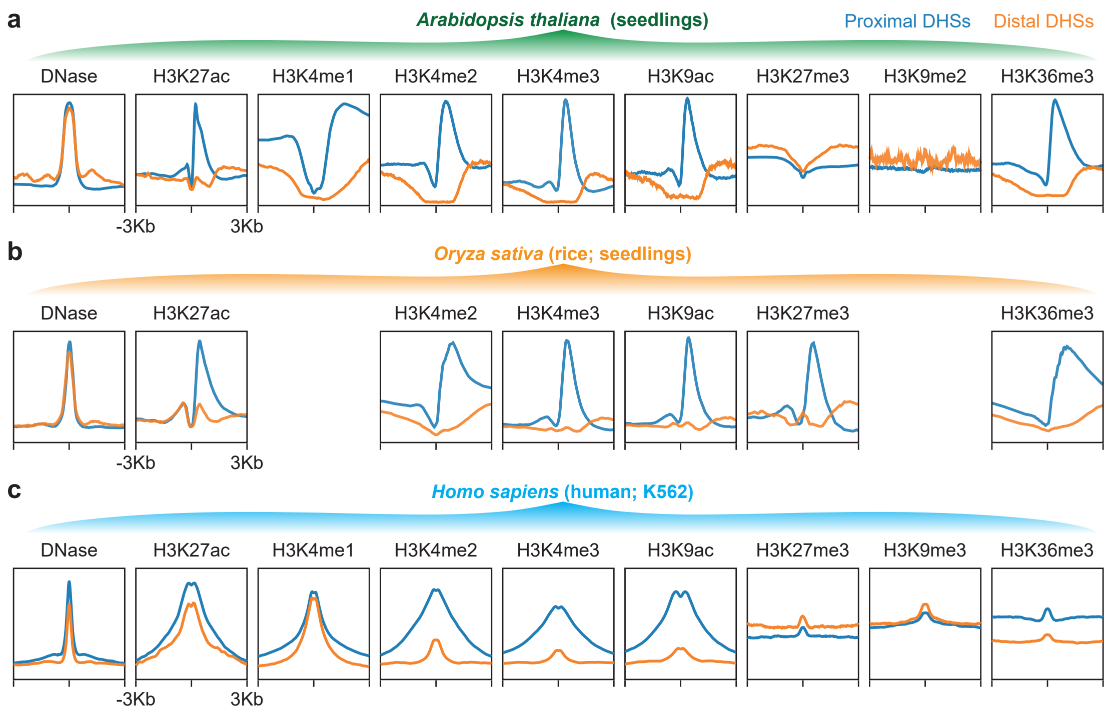

Supplementary Fig. 12: Histone modification ChIP intensity relative to the peak centers of proximal and distal DHSs (related to Figure 2d-f). Composite (average intensity) plots of various histone modification ChIP intensity relative to the peak centers of proximal and distal DHSs in Arabidopsis (a), rice (b) and human (c).

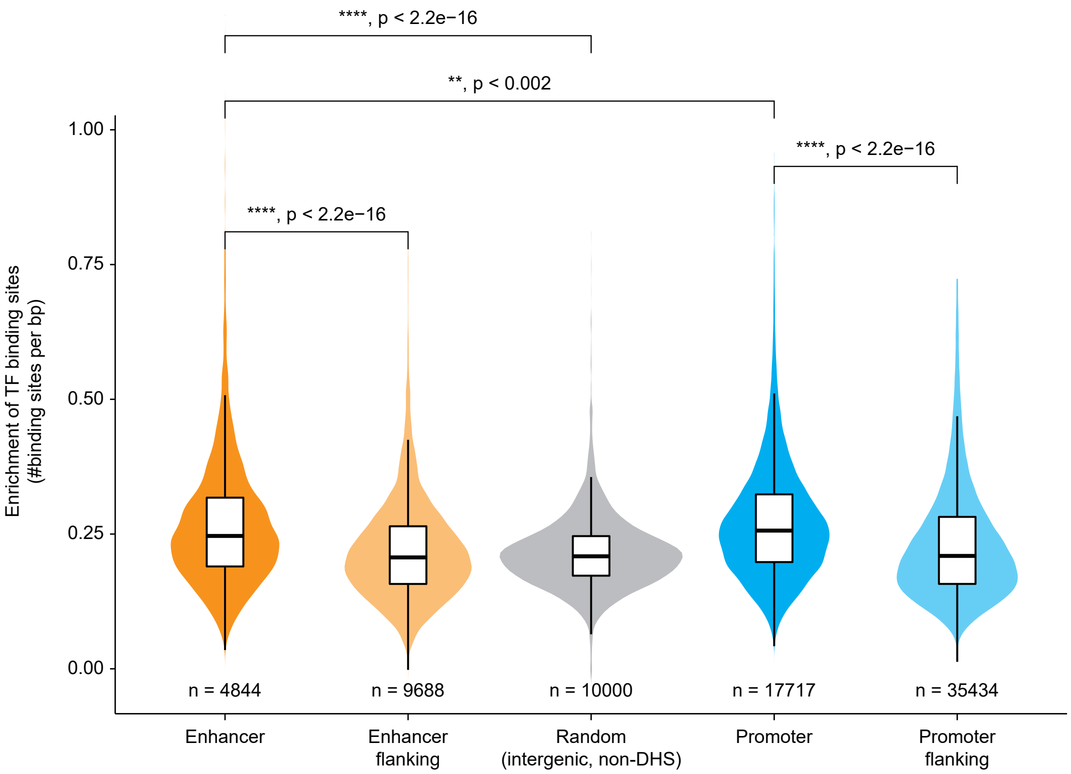

Supplementary Fig. 13: Enrichment of predicted transcription factor (TF) binding sites in putative distal enhancers. For comparisons, enrichment was also calculated in enhancer flanking regions (at both sides with a matched size), random intergenic non-DHS regions (n = 10000), promoters (proximal DHSs) and promoter flanking regions. Enrichment score was calculated as the number of TF biding sites per bp in each region. Significance was determined by Welch two sample t-test. DHSs, DNase I hypersensitive sites.

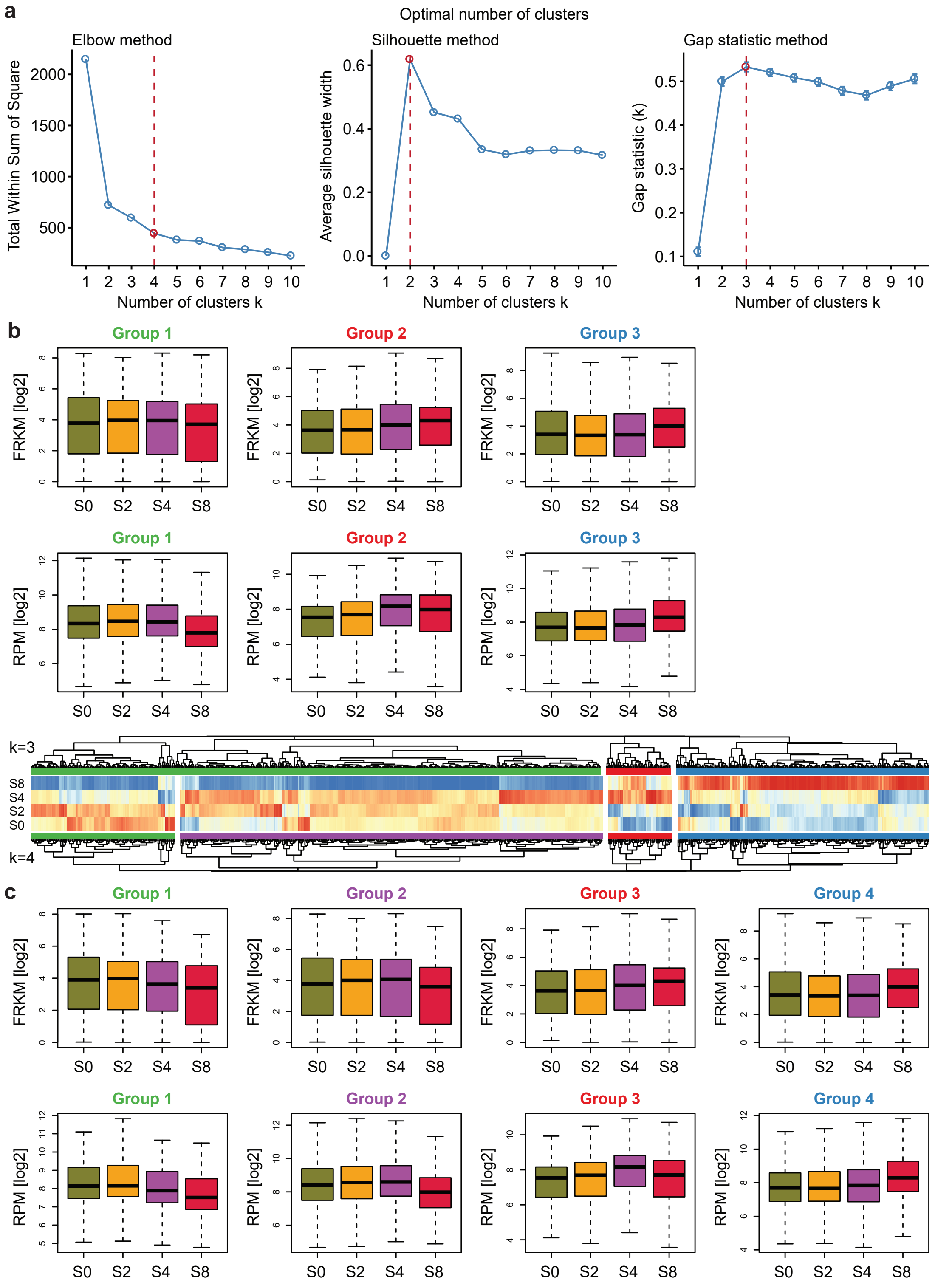

Supplementary Fig. 14: Enhancer dynamics across flower development (related to Figure 6). (a) Determining the optimal number of clusters using three different methods (elbow, Silhouette and gap statistic). The optimal number of clusters is highlighted in red. (b,c) Similar to **Figure 6a,b**, the enhancers are grouped into three (b) or four (c) classes. FPKM (fragments per kilo base per million mapped reads) for gene expression (top), and RPM (reads per million mapped reads) values for enhancer activity (bottom). From the boxplots, two major clusters are observed for clustering analysis with k=3 or k=4. Thus, we considered k=2 as the optimal number of clusters.

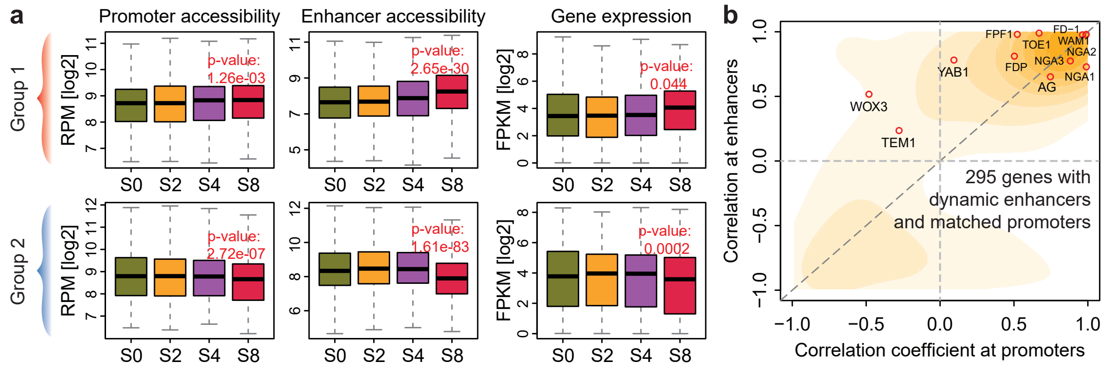

Supplementary Fig. 15: Correlation between chromatin accessibility and gene expression (related to Figure 6). (a) Boxplots showing the promoter accessibility, enhancer accessibility and corresponding gene expression patterns across developmental stages (from stage 0 [S0] to S8) for the genes in the two groups as indicated in **Figure 6a**. P-values were calculated based on Wilcoxon signed-rank paired tests. (b) Contour plot showing Pearson correlation coefficients between gene expression and chromatin accessibility at promter and enhancer regions. Only genes (N=295) with dynamic enhancers (in **Figure 6a**) and matched promters were used in the analysis.

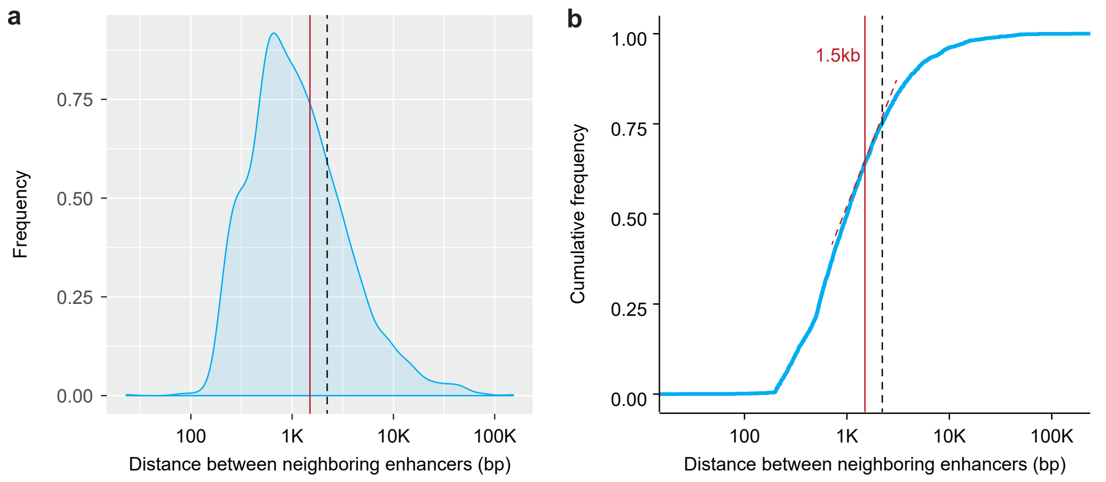

Supplementary Fig. 16: Determine the stitching distance used to identify enhancer clusters. (a) Density plot showing the distribution of distance between two neighboring enhancers within a same intergenic region. (b) Cumulative density plot showing the distribution of distance between two neighboring enhancers within a same intergenic region. The red line indiates the cutoff of stitching distance used for the identification of enhancer clusters. The dash line indates the average distance between two intergenic regions.

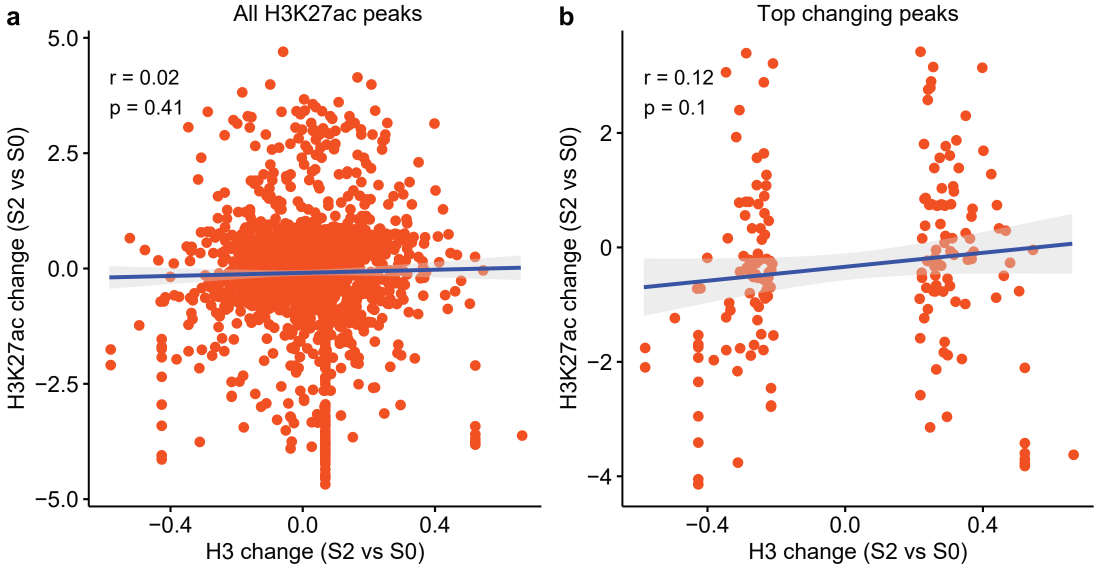

Supplementary Fig. 17: The dynamics of H3K27ac is independent on the dynamics of H3. (a) Correlation of the change of H3 levels and H3K27ac levels between stages 0 (S0) and 2 (S2). All H3K27ac peak regions were used in the analysis. (b) Similar to (a). Only the top regions (n=177) with highest changing H3 and H3K27ac were used in the analysis.

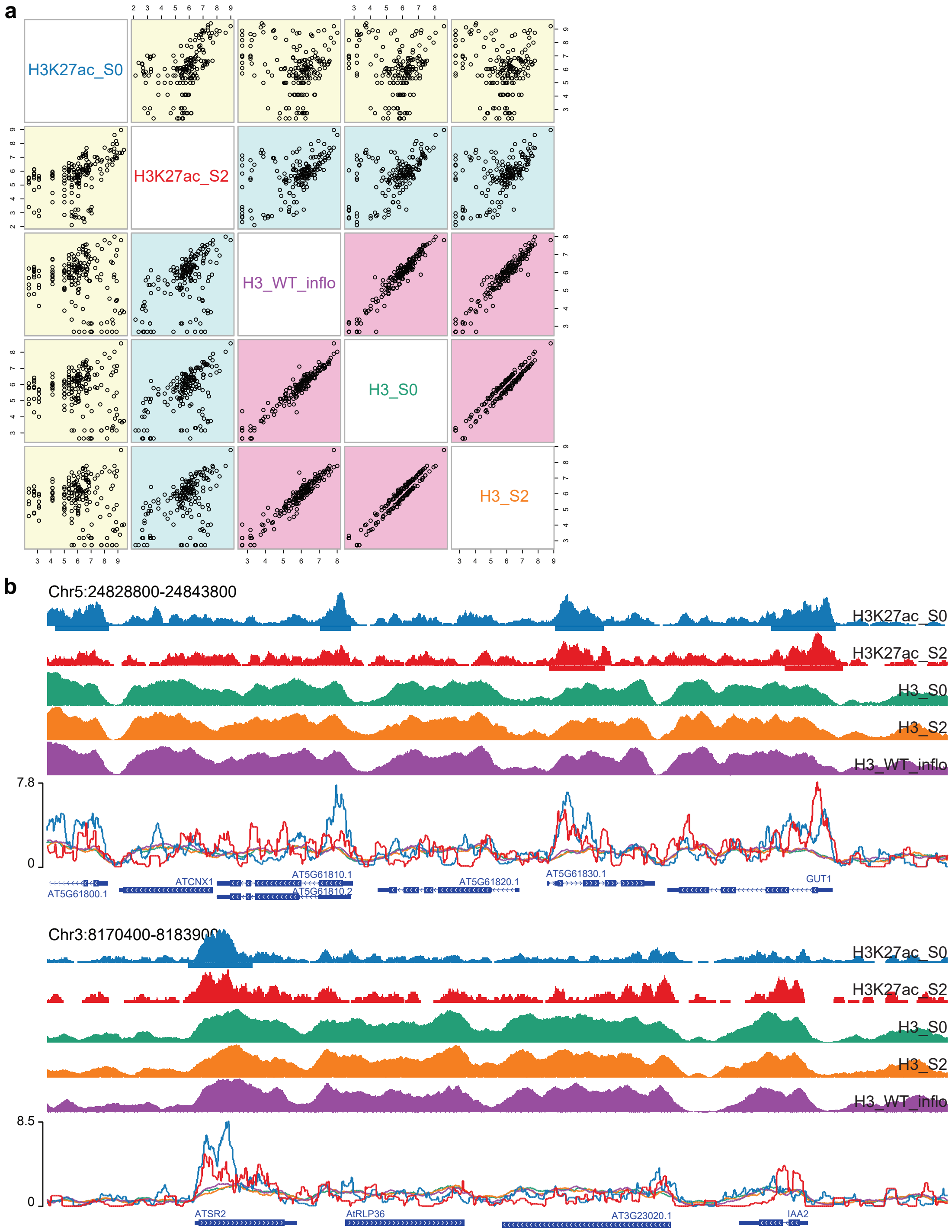

Supplementary Fig. 18: Comparison of H3K27ac and H3 ChIP-seq data. (a) Scatterplots showing the relationship of H3 and H3K27ac signals at stages 0 (S0) and 2 (S2; *ap1cal* pAP1::AP1-GR). H3 data in wildtype inflorescences (WT inflo) were used for comparison. Only the top regions (n=177) with highest changing H3 and H3K27ac were shown. (b) Genome browser views of H3K27ac and H3 signal at selected loci.

Supplementary Tables

Supplementary Table 1: DNA motif models for *Arabidopsis thaliana* transcription factors (TFs)

**Data source**	**Evidence**[1]	**#motifs/#TFs**[2]	**Motif ID with**[3]	**Reference**
JASPAR	ChIP-chip; ChIP-seq; PBM; SELEX	187/186	Jaspar_	ref.[1]
Athamap	-	139/94	Athamap_	ref.[2]
UniPROBE	PBM	36/16	UniPROBE_	ref.[3]
Sullivan et al. (2014)	PBM	46/43	cellrep_	ref.[4]
Weirauch et al. (2014)	SELEX; ChIP-seq	333/359	CISBP_	ref.[5]
Franco-Zorrilla et al. (2014)	PBM	108/64	PNAS_	ref.[6]
O'Malley et al. (2016)	DAP-seq	872/534	DAPseq_	ref.[7]
Summary	-	**1721/763**	-	-

[1] PBM: protein binding microarray; SELEX: Systematic evolution of ligands by exponential enrichment;

[2] #, the number of;

[3] The prefixes of motif IDs used in **Supplementary Data 2** and **5**.

Supplementary References

1. Mathelier, A. *et al.* JASPAR 2014: an extensively expanded and updated open-access database of transcription factor binding profiles. *Nucleic acids research* **42,** D142–D147 (2013).
2. Steffens, N. O., Galuschka, C., Schindler, M., Bülow, L. & Hehl, R. AthaMap web tools for database-assisted identification of combinatorial cis-regulatory elements and the display of highly conserved transcription factor binding sites in Arabidopsis thaliana. *Nucleic acids research* **33,** W397–W402 (2005).
3. Hume, M. A., Barrera, L. A., Gisselbrecht, S. S. & Bulyk, M. L. UniPROBE, update 2015: new tools and content for the online database of protein-binding microarray data on protein–DNA interactions. *Nucleic acids research* **43,** D117–D122 (2014).
4. Sullivan, A. M. *et al.* Mapping and dynamics of regulatory DNA and transcription factor networks in A. thaliana. *Cell reports* **8,** 2015–2030 (2014).
5. Weirauch, M. T. *et al.* Determination and inference of eukaryotic transcription factor sequence specificity. *Cell* **158,** 1431–1443 (2014).
6. Franco-Zorrilla, J. M. *et al.* DNA-binding specificities of plant transcription factors and their potential to define target genes. *Proceedings of the National Academy of Sciences* **111,** 2367–2372 (2014).
7. O'Malley, R. C. *et al.* Cistrome and epicistrome features shape the regulatory DNA landscape. *Cell* **165,** 1280–1292 (2016).
