# Supplementary material for: Dynamic control of enhancer activity drives stage-specific gene expression during flower morphogenesis
Source: Nat Commun. 2019 Apr 12;10:1705. doi: 10.1038/s41467-019-09513-2 (PMC6461659; doi:10.1038/s41467-019-09513-2)
Supplement: Supplementary file 3 — Description of Additional Supplementary Files [file 41467_2019_9513_MOESM3_ESM.pdf]

Description of Additional Supplementary Files

File Name: Supplementary Data 1
Description: The list of H3K27ac peaks

File Name: Supplementary Data 2
Description: H3K27ac dynamics during flower development

File Name: Supplementary Data 3
Description: The list of DHS-predicted intergenic enhancers

File Name: Supplementary Data 4
Description: Enhancer validation by GUS experiments

File Name: Supplementary Data 5
Description: Enhancer dynamics during flower development

File Name: Supplementary Data 6
Description: The list of intragenic enhancers

File Name: Supplementary Data 7
Description: The list of enhancer clusters
