# Supplementary material for: Dynamic control of enhancer activity drives stage-specific gene expression during flower morphogenesis
Source: Nat Commun. 2019 Apr 12;10:1705. doi: 10.1038/s41467-019-09513-2 (PMC6461659; doi:10.1038/s41467-019-09513-2)
Supplement: Supplementary file 11 — Reporting Summary [file 41467_2019_9513_MOESM11_ESM.pdf]

nature research

Corresponding author(s): Dr. Dijun Chen & Dr. Kerstin Kaufmann

Last updated by author(s): Feb 24, 2019

Reporting Summary

Nature Research wishes to improve the reproducibility of the work that we publish. This form provides structure for consistency and transparency in reporting. For further information on Nature Research policies, see Authors & Referees and the Editorial Policy Checklist.

Statistics

For all statistical analyses, confirm that the following items are present in the figure legend, table legend, main text, or Methods section.

n/a	Confirmed	
☐	☒	The exact sample size (*n*) for each experimental group/condition, given as a discrete number and unit of measurement
☐	☒	A statement on whether measurements were taken from distinct samples or whether the same sample was measured repeatedly
☐	☒	The statistical test(s) used AND whether they are one- or two-sided<br>*Only common tests should be described solely by name; describe more complex techniques in the Methods section.*
☒	☐	A description of all covariates tested
☐	☒	A description of any assumptions or corrections, such as tests of normality and adjustment for multiple comparisons
☒	☐	A full description of the statistical parameters including central tendency (e.g. means) or other basic estimates (e.g. regression coefficient) AND variation (e.g. standard deviation) or associated estimates of uncertainty (e.g. confidence intervals)
☐	☒	For null hypothesis testing, the test statistic (e.g. *F*, *t*, *r*) with confidence intervals, effect sizes, degrees of freedom and *P* value noted<br>*Give P values as exact values whenever suitable.*
☒	☐	For Bayesian analysis, information on the choice of priors and Markov chain Monte Carlo settings
☒	☐	For hierarchical and complex designs, identification of the appropriate level for tests and full reporting of outcomes
☒	☐	Estimates of effect sizes (e.g. Cohen's *d*, Pearson's *r*), indicating how they were calculated

Our web collection on statistics for biologists contains articles on many of the points above.

Software and code

Policy information about availability of computer code

Data collection	No special software was used for data collection.
Data analysis	The software used to analyze the data in this study are publicly available.<br><br>>> RNA-seq<br>RNA-seq reads were mapped to A. thaliana reference genome (TAIR10) using STAR (version 020201). Expression levels (in terms of FPKM, fragments per kilobase of transcript per million mapped reads) of all annotated protein-coding genes were estimated by RSEM (version 1.2.22). FPKM (fragments per kilobase of transcript per million mapped reads) values as defined by RSEM were added a pesudo-value of 1e-6 (to avoid zeros) and then log2-transformed. A gene was considered as expressed only its estimated FPKM > 0.1 at least one sample. Differentially expressed genes across the four developmental stages were identified by analysis of variance (ANOVA) based on FPKM values. Resulting p-values were adjusted for multiple comparisons by false discovery rate (FDR). Genes were considered as differentially expressed if they showed at least two-fold changes with FDR < 0.05.<br><br>>> ChIP-seq<br>The quality of the raw data (FASTQ files) was evaluated by FastQC (http://www.bioinformatics.babraham.ac.uk/projects/fastqc/). Reads were then mapped to the A.thaliana genome (TAIR10) using Bowtie (version 1.1.2) with parameters "--threads 8 -n 2 -m 3 -k 1 --best --chunkmbs 256 -q". Redundant reads were removed using Picard tools (v2.60; http://broadinstitute.github.io/picard/). Peak calling was performed using MACS2 (version 2.1.0). Duplicated reads were not considered (--keep-dup=1) during peak calling in order to achieve a better specificity. The "--mfold" parameter was set as "2-20" to build the model. The parameter "-broad" was used in the calling of H3K27me3-enriched regions (broad peaks), whereas '--call-summits' was used in the calling of narrow peaks for REF6 ChIP-seq data (see below). A relaxed threshold of p-value (p-value ≤ 1e-2) was suggested in order to enable the correct computation of IDR (irreproducible discovery rate) values. Following the recommendations for the analysis of self-consistency and reproducibility between replicates (https://sites.google.com/site/anshulkundaje/projects/idr), control samples were combined into one single control among the replicated experiments. Peaks across replicates with an IDR ≤ 0.05 were retained. For visualization purpose, wiggle tracks (using pooled data across biological replicates) were generated by DeepTools with the command "bamCoverage"; read coverage was normalized as RPKM (Reads

Per Kilobase per Million reads).

>> Statistics and data visualization
R (https://cran.r-project.org/; version 3.2.3) was used to compute statistics and generate plots. ChIP-seq data signal tracks were visualized in the WashU Epigenome Browser.

For manuscripts utilizing custom algorithms or software that are central to the research but not yet described in published literature, software must be made available to editors/reviewers. We strongly encourage code deposition in a community repository (e.g. GitHub). See the Nature Research guidelines for submitting code & software for further information.

Data

Policy information about availability of data

All manuscripts must include a data availability statement. This statement should provide the following information, where applicable:

- Accession codes, unique identifiers, or web links for publicly available datasets
- A list of figures that have associated raw data
- A description of any restrictions on data availability

ChIP-seq data generated in this study data have been submitted to the NCBI Gene Expression Omnibus (GEO; http://www.ncbi.nlm.nih.gov/geo/) under accession number GSE112965. The RNA-seq data derived during this study are available under GEO accession number GSE110500. The source data underlying Figs. 1d, 1e, 2a, 2b, 2g, 3a, 3c, 3d, 3g-i, 4a, 4c, 5a, 5c, 6, 7a-c, 7e and 7f are provided in the Source Data file.

Field-specific reporting

Please select the one below that is the best fit for your research. If you are not sure, read the appropriate sections before making your selection.

☒ Life sciences ☐ Behavioural & social sciences ☐ Ecological, evolutionary & environmental sciences

For a reference copy of the document with all sections, see nature.com/documents/nr-reporting-summary-flat.pdf

Life sciences study design

All studies must disclose on these points even when the disclosure is negative.

Sample size	For all RNA-seq experiments, we collected flower tissues from 20 plants as one sample, and three independent collections were performed. For ChIP-seq, we harvested inflorescences from about 100 plants as one sample. Tissues were harvested two to three times from different batches of plants for two biological replicates.
Data exclusions	No data were excluded.
Replication	For each RNA-seq, experiments, we performed three biological replicates and for the ChIPseq, two biological replicates were performed. Data from all the attempts were presented and the correlation between replicates and quality of the data were high, as showed in the supporting data of the manuscript.
Randomization	Plants were grown in a completely randomized manner and the tray was rotated every two days when the plants were grown in the growth chamber.
Blinding	No blinding was used.

Reporting for specific materials, systems and methods

We require information from authors about some types of materials, experimental systems and methods used in many studies. Here, indicate whether each material, system or method listed is relevant to your study. If you are not sure if a list item applies to your research, read the appropriate section before selecting a response.

Materials & experimental systems

n/a	Involved in the study	
☐	☒	Antibodies
☒	☐	Eukaryotic cell lines
☒	☐	Palaeontology
☒	☐	Animals and other organisms
☒	☐	Human research participants
☒	☐	Clinical data

Methods

n/a	Involved in the study	
☐	☒	ChIP-seq
☒	☐	Flow cytometry
☒	☐	MRI-based neuroimaging

Antibodies

Antibodies used	All antibodies used in this study are commercially available:

	anti-H3K27ac (Lot.2322526, Millipore, Germany);<br>anti-H3 (ab1797, abcam, UK)
Validation	Specificity of the anti-H3K27ac antibody was verified prior to its usage (see Supplementary Fig. 1).

ChIP-seq

Data deposition

☒ Confirm that both raw and final processed data have been deposited in a public database such as GEO.

☒ Confirm that you have deposited or provided access to graph files (e.g. BED files) for the called peaks.

Data access links<br>*May remain private before publication.*	https://www.ncbi.nlm.nih.gov/geo/query/acc.cgi?&acc=GSE112965
Files in database submission	GSE112965_H3K27ac_day0.narrowPeak.bed.gz<br>GSE112965_H3K27ac_day2.narrowPeak.bed.gz<br>GSE112965_H3K27ac_day4.narrowPeak.bed.gz<br>GSE112965_H3K27ac_day8.narrowPeak.bed.gz<br>GSM3092473_H3K27ac-Control.rpgc.bw<br>GSM3092475_H3K27ac_day0_Rep1.rpgc.bw<br>GSM3092476_H3K27ac_day0_Rep2.rpgc.bw<br>GSM3092477_H3K27ac_day2_Rep1.rpgc.bw<br>GSM3092478_H3K27ac_day2_Rep2.rpgc.bw<br>GSM3092479_H3K27ac_day4_Rep1.rpgc.bw<br>GSM3092480_H3K27ac_day4_Rep2.rpgc.bw<br>GSM3092481_H3K27ac_day8_Rep1.rpgc.bw<br>GSM3092482_H3K27ac_day8_Rep2.rpgc.bw
Genome browser session<br>(e.g. UCSC)	NA

Methodology

Replicates	2 biological replicates
Sequencing depth	The average read number for each single replicate is ca. 21M, of which 33-55% unique mapping. Read length: 76bp in single-end.
Antibodies	IP antibody for H3K27ac: anti-H3K27ac (Lot.2322526, Millipore, Germany)<br>H3: anti-H3 (ab1797, abcam, UK)
Peak calling parameters	Reads were mapped to the A.thaliana genome (TAIR10) using Bowtie (version 1.1.2) with parameters "--threads 8 -n 2 -m 3 -k 1 --best --chunkmbs 256 -q". Redundant reads were removed using Picard tools (v2.60; http://broadinstitute.github.io/picard/). Peak calling was performed using MACS2 (version 2.1.0). Duplicated reads were not considered (--keep-dup=1) during peak calling in order to achieve a better specificity. The "--mfold" parameter was set as "2-20" to build the model. The parameter '--call-summits' was used in the calling of narrow peaks for H3K27ac ChIP-seq data. Peaks across replicates with an IDR ≤ 0.05 (https://sites.google.com/site/anshulkundaje/projects/idr) were retained.
Data quality	In our analysis, we used H3K27ac peaks at FDR < 5%, and > 35% of the H3K27ac peaks above 4-fold enrichment.
Software	The quality of the raw data (FASTQ files) was evaluated by FastQC (http://www.bioinformatics.babraham.ac.uk/projects/fastqc/). Wiggle tracks were generated by DeepTools with the command "bamCoverage"; read coverage was normalized as RPKM (Reads Per Kilobase per Million reads). ChIP-seq data signal tracks were visualized in the WashU Epigenome Browser.
